# A study on the pharmacovigilance of various SGLT-2 inhibitors

**DOI:** 10.3389/fmed.2024.1515847

**Published:** 2025-01-15

**Authors:** Yanwen Dong, Yangyang Wang, Xiaomei Lan, Huiyan Zeng

**Affiliations:** ^1^Guangzhou University of Chinese Medicine, Guangzhou, China; ^2^The Second Clinical College of Guangzhou University of Chinese Medicine, Guangzhou, China; ^3^The Affiliated Huizhou Hospital, Guangzhou Medical University, Guangzhou, China; ^4^Guangzhou Development District Hospital, Guangzhou, China; ^5^The Second Affiliated Hospital of Guangzhou University of Chinese Medicine, Guangzhou, China

**Keywords:** SGLT2i, adverse reactions, safe medication, pharmacovigilance, disproportionality analysis

## Abstract

**Background:**

Sodium-glucose co-transporter two inhibitors (SGLT2is) are widely used in clinical practice due to their proven cardiovascular and renal benefits. However, various adverse drug reactions (ADRs) have been reported. This study aims to systematically update the ADRs associated with SGLT2is and identify the differences among various SGLT2is acovigilance of various SGLT-2 inhibitors.

**Methods:**

Data from the FAERS database covering Q1 2013 to Q2 2024 were selected for disproportionality analysis. ADRs were defined using the System Organ Classes (SOC) and Preferred Terms (PT) from the MedDRA 27.0 dictionary. Four signal detection metrics—reporting odds ratio (ROR), proportional reporting ratios (PRRs), Bayesian Confidence Propagation Neural Network (BCPNN), and empirical Bayesian geometric mean (EBGM)—were utilized to infer ADRs and assess differences among specific SGLT2i drugs through intersection analysis.

**Results:**

Except for canagliflozin, both dapagliflozin and empagliflozin showed a general increase in ADRs. Specifically, canagliflozin had 93 ADRs, dapagliflozin had 173, and empagliflozin had 214. Most of these were related to Infections and Infestations, Investigations, and Reproductive System and Breast Disorders, notably manifesting as inflammatory conditions of the urinary and reproductive systems, such as orchitis and testicular abscess, consistent with FDA labeling. Additionally, overlooked ADRs were identified, including bladder cancer, cholangiocarcinoma, and thrombotic strokes, none of which were reported for canagliflozin.

**Discussion:**

While shared ADRs for SGLT2is are noted in FDA labeling, monitoring for high-risk populations, such as those with cancers or strokes, remains crucial to prevent deterioration. Medication regimens may need adjustment, including selecting canagliflozin or non-SGLT2i alternatives when necessary.

## 1 Introduction

With rapid economic growth, an increase in unhealthy diets, and sedentary lifestyles, there has been a significant rise in body mass index (BMI) and fasting plasma glucose levels. Consequently, the burden of diabetes is escalating, with studies indicating that over 500 million people are currently affected worldwide, resulting in a prevalence rate of 6.1% (1). This condition not only impacts individual functionality and quality of life but also leads to premature severe mortality (2). Survivors often face complications such as kidney damage. Sodium-glucose co-transporter two inhibitors (SGLT2is) complement the action of RAAS inhibitors by restoring the tubuloglomerular feedback mechanism, improving local hypoxia, and enhancing autophagy. These effects lower blood sugar levels and effectively prevent the progression of diabetic kidney disease (3), establishing SGLT2is as a “rising star” among antidiabetic medications.

Unfortunately, as the use of SGLT2is has expanded and observation periods have lengthened, researchers and clinicians have identified some unexpected adverse reactions. While the FDA labeling clearly outlines risks associated with SGLT2is, such as hypoglycemia, genital infections, and amputations (4), Gong et al. (5) reported a case of abnormal uterine bleeding attributed to dapagliflozin despite adherence to clinical guidelines. Furthermore, Xu et al. (6) suggested a potential risk of renal cancer linked to SGLT2is. These concerning ADRs have led to increased discontinuation rates (7), significantly affecting the implementation of prescribed treatment plans.

Therefore, systematically understanding the potential adverse drug reactions (ADRs) of different SGLT2 inhibitors and their differences is of significant importance for improving diabetes mellitus (DM) and its related complications. Although researchers have paid attention to clinical issues related to SGLT-2 inhibitors, their studies have often focused on specific types of adverse reactions, such as Zhao, B. (8)‘s study on the risk of fractures caused by SGLT-2 inhibitors. However, a comprehensive overview of ADRs associated with different SGLT-2 s and the differences in adverse reactions between different SGLT-2 inhibitors have not received adequate attention. Although there are studies analyzing the ADRs of individual drugs (9), these studies struggle to provide convincing comparative results due to the lack of detection methods and assessment standards. With the growing demand for precision medicine and personalized medication, a comprehensive understanding and emphasis on these differences are particularly crucial, which is also the direction our research is committed to supplementing and improving. On the other hand, as drug adverse events accumulate and update, the results obtained from these previous studies, as well as those more potential ADRs, urgently require more timely and accurate research reports for clarification and identification. To this end, FAERS (FDA Adverse Event Reporting System) provides possibilities. This study employs data mining techniques to search the most recently updated FAERS database, conduct disproportionality analysis to detect, analyze, and update SGLT2 inhibitor safety events, and compare the similarities and differences in ADRs between different SGLT2 inhibitors, promoting the rational use of drugs.

## 2 Methods

### 2.1 Data source

This observational analysis utilizes the FDA Adverse Event Reporting System (FAERS) database, which contains over 20 million ADR reports from the United States, Europe, and Asia. Reports are submitted by both healthcare professionals (such as doctors, pharmacists, and registered nurses) and non-healthcare individuals (including consumers, lawyers, and vendors). Each report includes a unique identifier, patient demographics (such as gender, age, and weight), report date, report’s country, qualifications of the primary reporter, suspected medications and their indications, ADR occurrence date, severity, and a detailed description of each ADR. Additionally, ADR descriptions are coded using Preferred Terms (PT) from the MedDRA® version 27.0 and categorized by System Organ Classes (SOC). The dataset is updated quarterly, facilitating the timely identification of ADRs that may not have been detected before market release.

SGLT-2 inhibitors were first approved for use in 2013. To conduct post-marketing surveillance, this study spans from the first quarter of 2013 to the second quarter of 2024, collecting relevant reports on SGLT-2 inhibitors, including canagliflozin, dapagliflozin, and empagliflozin, while ensuring the removal of duplicates. To address the lack of standardization in drug naming, our research team gathered generic and brand names for SGLT-2 inhibitors and manually screened the reports. We excluded other medications, such as atorvastatin, metoprolol, and hydrochlorothiazide, to minimize the risk of false positives. To enhance the professionalism and reliability of our findings, we only included reports submitted by healthcare professionals, such as doctors and pharmacists, that identified SGLT-2 inhibitors as the primary suspected agents. Two researchers conducted the data organization process independently, with a third researcher making the final decision in case of any discrepancies.

### 2.2 Data analysis

This study assessed demographic and clinical characteristics from multiple dimensions, including gender, age groups, reporter types, and reporting countries. Based on the principle of signal disproportionality analysis, to ensure the reliability of the results, we combined four risk signal detection methods in this study, including: ROR, PRR, EBGM, and BPCNN (10, 11). The Reporting Odds Ratio (ROR) is a widely used signal detection tool in the field of pharmacovigilance, which assesses the potential association between a specific drug and a specific adverse event (AE) by calculating the reporting odds ratio. An ROR value greater than 1 indicates that the association between the drug and the adverse event may be greater than random, and the higher the value, the stronger the association. The advantage of ROR is its simple and intuitive calculation method, which is easy to understand and apply, especially suitable for preliminary signal detection in large databases. In addition, the threshold setting of ROR is crucial for identifying potential safety signals, with common thresholds including ROR values greater than 2 or higher, which are generally considered the starting point for signal detection and require further assessment and verification (12). The Proportional Reporting Ratio (PRR) is similar to ROR and is also a method for assessing the association between drugs and adverse events, but its calculation is based on the proportion of reports rather than absolute numbers. PRR compares the reporting proportion of a specific drug with a specific adverse event to the reporting proportion of the adverse event among all drugs to assess the association between the two. The advantage of PRR is its ability to better control the impact of different drug usage frequencies, making comparisons between different drugs more fair and accurate. The threshold setting of PRR is equally important, as it helps to identify adverse events that are disproportionately reported, providing a basis for further analysis and research (13). The Empirical Bayes Geometric Mean (EBGM) is a signal detection method based on Bayesian theory, which detects potential signals by empirical Bayesian shrinkage estimation of reported data. The advantage of EBGM is its ability to handle rare events and small sample data, providing more robust signal detection results. This method adjusts the random fluctuations in the data, making signal detection more stable and reliable. The threshold setting of EBGM, such as EBGM values greater than 2 or 3, helps to identify signals with statistical significance that require further attention and research (14). The Bayesian Confidence Propagation Neural Network (BPCNN) is a signal detection method that combines Bayesian theory and artificial neural networks. BPCNN can handle the multivariate relationships between drugs and adverse events through complex probability models, especially suitable for the analysis of high-dimensional data. The advantage of BPCNN is its ability to quantify uncertainty and improve the sensitivity and specificity of signal detection. The threshold setting of BPCNN, such as a Bayesian factor greater than 10, helps to identify adverse events with strong signals that may require immediate attention and intervention (15). The calculation methods and detection criteria of different methods in this study are shown in Table 1. It is generally believed that the combination of four risk signal detections has a higher level of evidence support (16). To ensure the accuracy of the results of this study, an adverse event is defined as a positive adverse event of SGLT-2 inhibitors only when all four algorithms are met.

**Table 1:** Disproportionality analysis formulas and criteria.

	Target drug	Not-target drug
Target ADR	a	c
Not-target ADR	b	d
N = a + b + c + d		
ROR = (ad)/(bc)	PRR = [a(c + d)]/[c(a + b)]	
95%CI = eln(ROR) + 1.96√(1/a + 1/b + 1/c + 1/d)	X^2^ = N(ad-bc)2/[(a + b)(c + d)(a + c)(b + d)]	
The criteria of positive safety signal detection by ROR:the lowerlimit of 95%Cl > 1,a ≥ 3	The criteria of positive safety signal detection by PRR: PRR ≥ 2，X^2^ ≥ 4，N ≥ 3	
IC = log2(aN)/(a + b)(a + c)	EBGM = (aN)/[(a + b)(a + c)]	
95%CI = E(IC) ± 2*√V(IC)	95%CI = eln(EGBM) ± 1.96√(1/a + 1/b + 1/c + 1/d)	
The criteria of positive safety signal detection of BPCNN:IC025 > 0(IC025:the lower bound of 95%CD)	The positive safety signal detection criteria by EGBM:EBGM05 > 2(EBGM05:the lower bound of 95%CI)	

In addition, the impact of drugs may have population biases. To exclude the impact of potential confounding factors (such as age, gender, weight, and reporting time), we also conducted a multivariate logistic regression analysis. Medication information is used as a characteristic variable (those taking the target drug are 1, otherwise 0), and positive adverse events are used as binary outcome variables (those occurring are 1, otherwise 0). Through this method, we can more accurately assess the association between drugs and positive ADRs, whether it is independent of confounding factors (such as age, gender, weight, and reporting time).

Subsequently, we conducted intersection analysis to identify common and unique ADRs of different SGLT-2 inhibitors, which helps to identify common safety risks within the drug class, optimize drug selection (such as choosing drugs that cause relatively fewer adverse events) to improve treatment efficacy and patient compliance, and promote future clinical research on common ADRs, support health regulatory departments to formulate or adjust drug use guidelines and policies, thereby providing precise basis for clinical medication selection. All statistical analyses of this study were conducted using R 4.3.2 software.

## 3 Results

### 3.1 Overall events and general characteristics

From 2013 to 2024, the FAERS database recorded a total of 8,731 adverse reactions related to canagliflozin, 9,417 related to dapagliflozin, and 15,848 related to empagliflozin (Table 1). Table 1 summarizes the clinical characteristics of cases associated with these three drugs. Among these adverse reactions, the incidence in females was lower than in males for all three medications (dapagliflozin ADR male: female = 50.1%: 40.6%; canagliflozin ADR male: female = 42.7%: 40.4%; empagliflozin ADR male: female = 50.4%: 36.5%). The age groups most affected by adverse reactions to these medications were 18–64 years and 65–85 years. Healthcare professionals reported all cases. For canagliflozin ADR reports, the top five reporting countries were the United States (76.9%), Canada (6.3%), Japan (6.2%), the United Kingdom (4.4%), and Spain (1.4%). For dapagliflozin ADR reports, the top five countries were the United States (32.7%), Japan (13.4%), China (9.6%), the United Kingdom (7.4%), and France (5.6%). For empagliflozin ADR reports, the top five countries were the United States (41.4%), Germany (7.5%), Japan (6.9%), the United Kingdom (6.2%), and Canada (4.4%).

**Figure 1:**
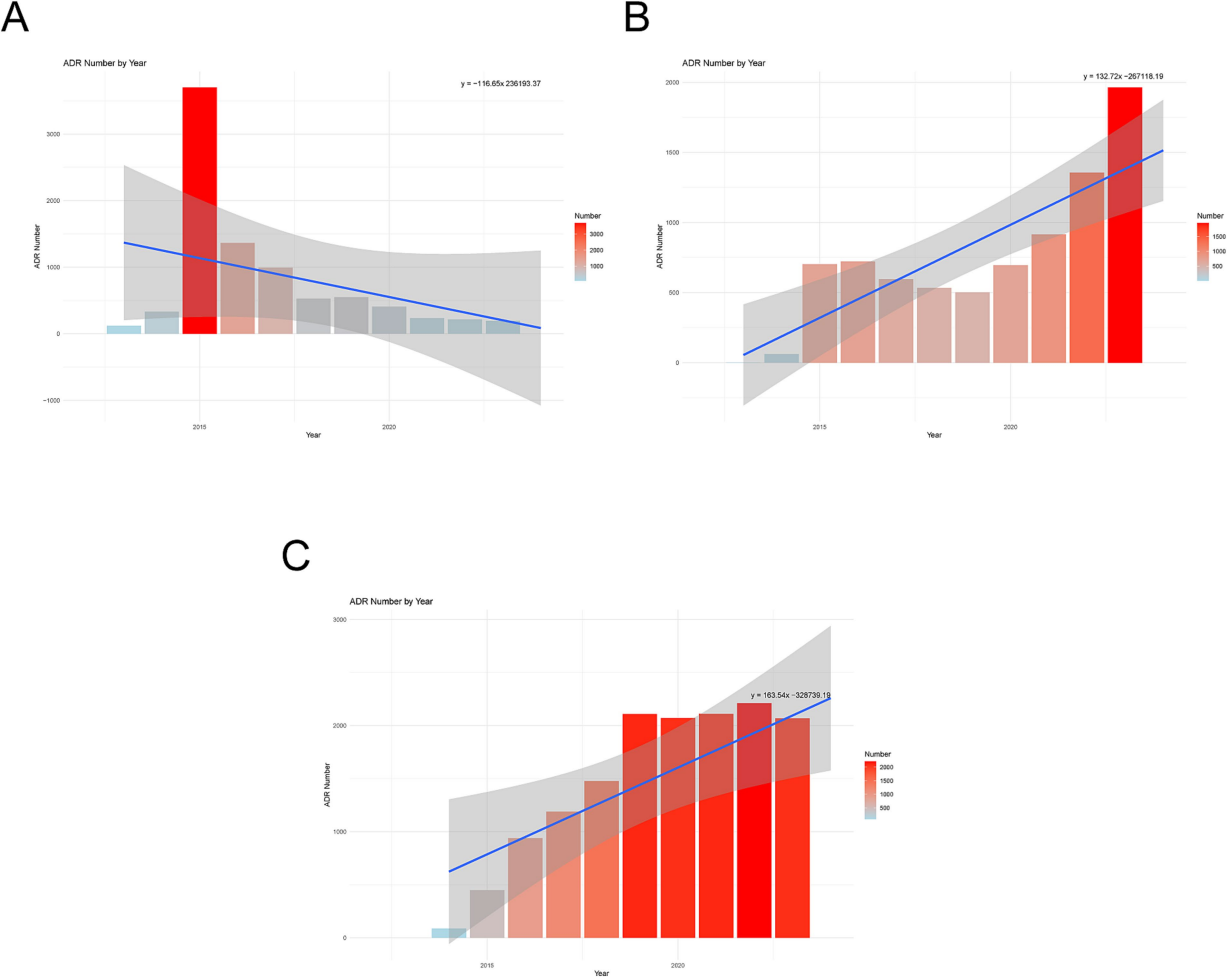
Annual trend chart of adverse drug reactions. **(A)** Canagliflozin. **(B)** Dapagliflozin. **(C)** Empagliflozin.

We created annual trend graphs (Figure 1) based on the ADRs reported for the drugs. The regression curves indicate that, except canagliflozin, both dapagliflozin and empagliflozin show a consistent upward trend in ADRs. Notably, canagliflozin (Figure 1A) displayed an increasing trend in the early stages; however, there was a sudden decline in reported cases after 2016. In contrast, ADR reports for dapagliflozin (Figure 1B) and empagliflozin (Figure 1C) continued to rise during the same period.

### 3.2 Identification of positive ADRs for canagliflozin

**Figure 2:**
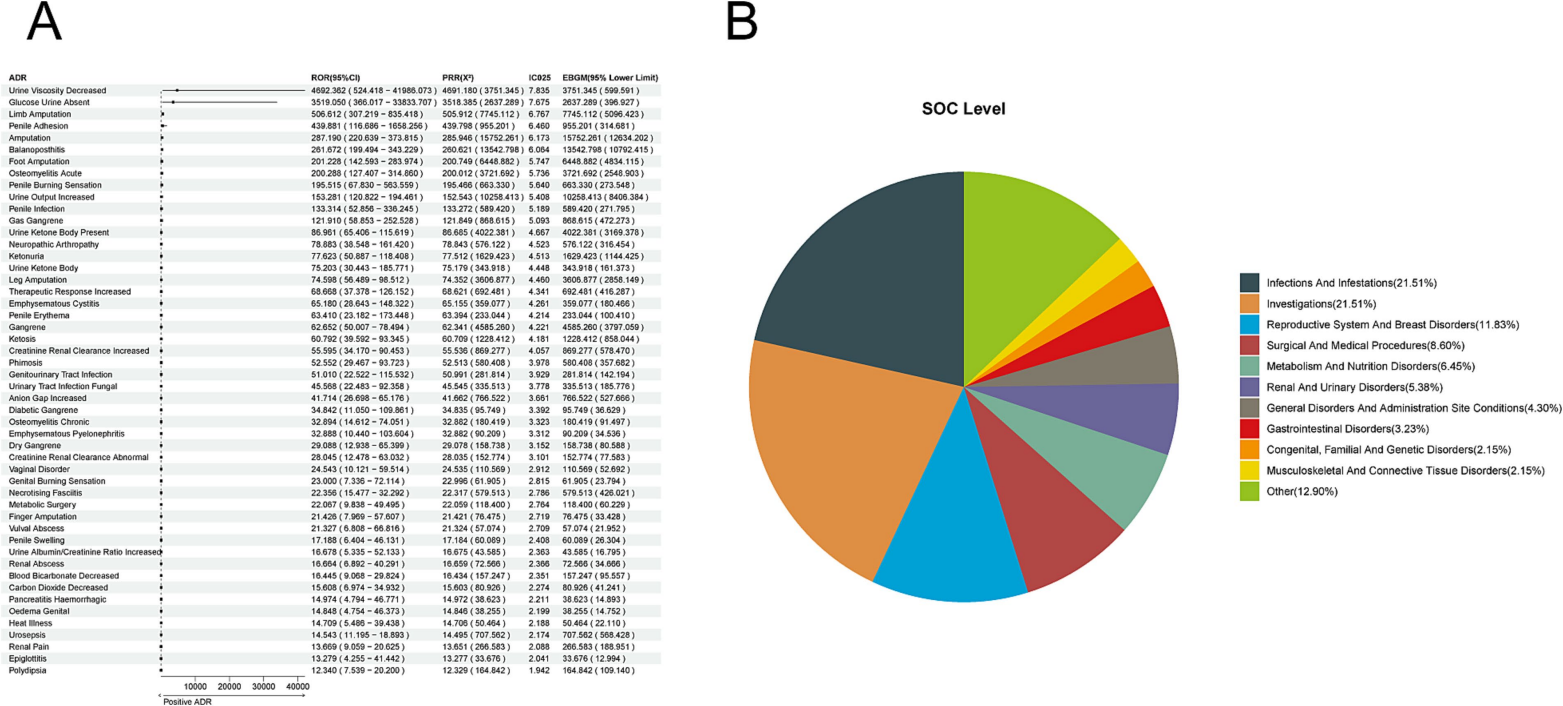
Positive ADR signals for Canagliflozin. **(A)** ROR top 50 forest graph of positive ADRs for canagliflozin. **(B)** Canagliflozin positive ADR System Organ Class (SOC) top 10 pie chart.

Based on 8,731 reports of adverse reactions associated with Canagliflozin from the overall SGLT-2 inhibitor events, we employed four methods to investigate further the adverse drug reactions (ADRs) related to canagliflozin. At the preferred term (PT) level, we identified 93 ADRs. The top five reactions based on the reporting odds ratio (ROR) were: Urine Viscosity Decreased [a:4, ROR (95% CI lower limit): 4692.362 (524.418), PRR (X^2^): 4691.180 (3751.345), IC025: 7.835, EBGM (95% CI lower limit): 599.591]; Glucose Urine Absent [a:3, ROR (95% CI lower limit): 3519.05 (366.017), PRR (X^2^): 3518.385 (2637.289), IC025: 7.675, EBGM (95% CI lower limit): 396.927]; Limb Amputation [a:22, ROR (95% CI lower limit): 506.612 (307.219), PRR (X^2^): 505.912 (7745.112), IC025: 6.767, EBGM (95% CI lower limit): 5096.423]; Penile Adhesion [a:3, ROR (95% CI lower limit): 439.881 (116.686), PRR (X^2^): 439.798 (955.201), IC025: 6.460, EBGM (95% CI lower limit): 314.681]; and Amputation [a:69, ROR (95% CI lower limit): 287.19 (220.639), PRR (X^2^): 285.946 (15752.261), IC025: 6.173, EBGM (95% CI lower limit): 12634.202] (Figure 2A). When mapping the ADRs of canagliflozin to the system organ class (SOC) level, the top five categories were Infections and Infestations (21.51%), Investigations (21.51%), Reproductive System and Breast Disorders (11.83%), Surgical and Medical Procedures (8.60%), and Metabolism and Nutrition Disorders (6.45%) (Figure 2B).

### 3.3 Identification of positive ADRs for dapagliflozin

**Figure 3:**
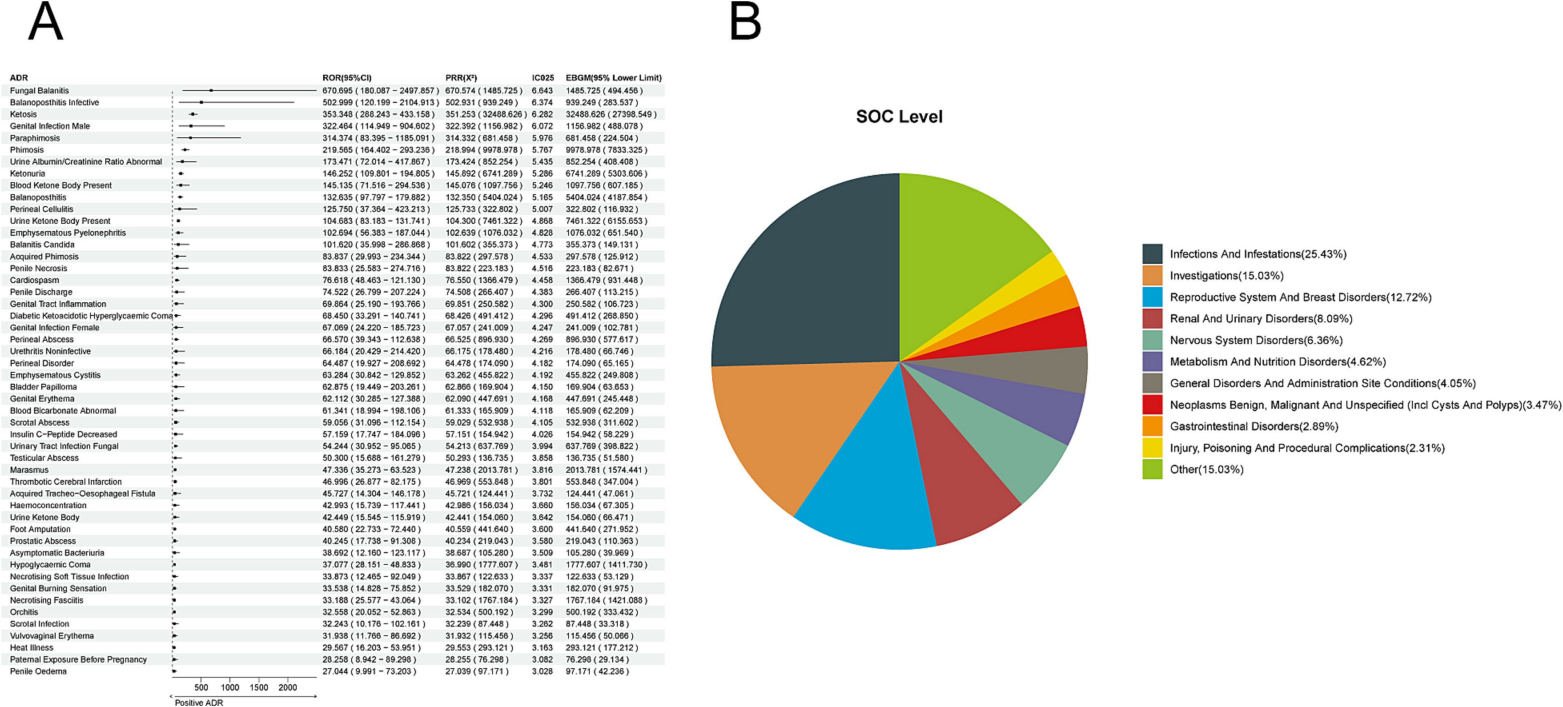
Positive ADR signals for dapagliflozin. **(A)** ROR top 50 forest graph of positive ADRs for dapagliflozin. **(B)** Dapagliflozin positive ADR System Organ Class (SOC) Top 10 pie chart.

We extracted 9,417 reports of dapagliflozin from the overall population of SGLT-2 inhibitor adverse event reports. We employed four methods to analyze further the ADRs associated with dapagliflozin, identifying 173 ADRs at the preferred term (PT) level. The top-ranked ADRs based on ROR included Fungal Balanitis [a:4, ROR (95% CI lower limit): 670.695 (180.087), PRR (X^2^): 670.574 (1485.725), IC025: 6.643, EBGM (95% CI lower limit): 494.456]; Balanoposthitis Infective [a:3, ROR (95% CI lower limit): 502.999 (120.199), PRR (X^2^): 502.931 (939.249), IC025: 6.374, EBGM (95% CI lower limit): 283.537]; Ketosis [a:132, ROR (95% CI lower limit): 353.348 (288.243), PRR (X^2^): 351.253 (32488.626), IC025: 6.282, EBGM (95% CI lower limit): 27398.549]; Genital et al. [a:5, ROR (95% CI lower limit): 322.464 (114.949), PRR (X^2^): 322.392 (1156.982), IC025: 6.072, EBGM (95% CI lower limit): 488.078]; and Paraphimosis [a:3, ROR (95% CI lower limit): 314.374 (83.395), PRR (X^2^): 314.332 (681.458), IC025: 5.976, EBGM (95% CI lower limit): 224.504] (Figure 3A). The corresponding System Organ Class (SOC) levels were primarily concentrated in Infections and Infestations (25.43%), Investigations (15.03%), Reproductive System and Breast Disorders (12.72%), Renal and Urinary Disorders (8.09%), and Nervous System Disorders (6.36%) (Figure 3B).

### 3.4 Identification of positive ADR signals for empagliflozin

**Figure 4:**
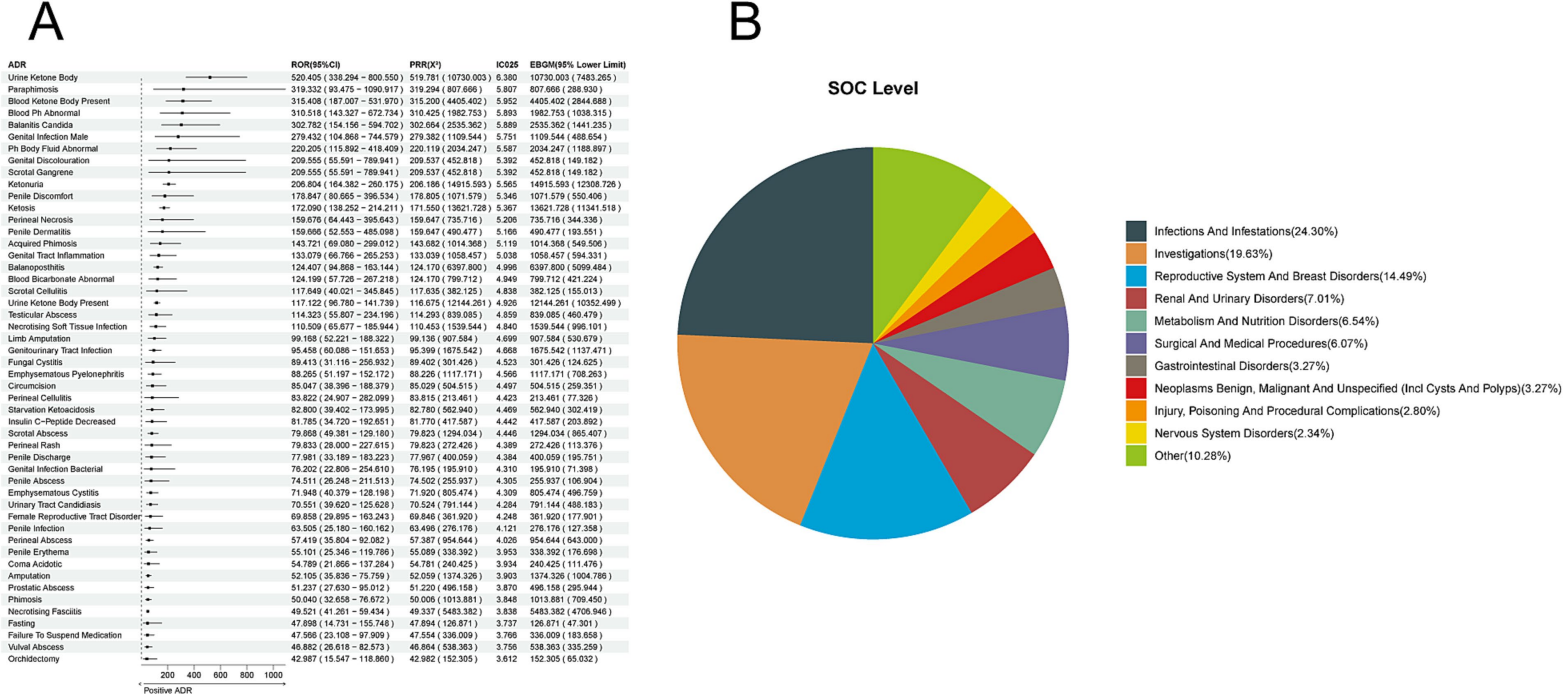
Positive ADR signals for empagliflozin. **(A)** ROR Top 50 forest graph of positive ADRs for empagliflozin. **(B)** Empagliflozin positive ADR System Organ Class (SOC) top 10 pie chart.

Based on 15,848 reports of adverse reactions related to all SGLT-2 inhibitors, we employed four methods to identify positive ADR signals for empagliflozin, identifying 214 ADRs at the Preferred Term (PT) level. The top five ADRs identified based on the Reporting Odds Ratio (ROR) were as follows: Urine Ketone Body [a:40, ROR (95% CI lower limit): 520.405 (338.294), PRR (X^2^): 519.781 (10,730.003), IC025: 6.380, EBGM (95% CI lower limit): 7483.265]; Paraphimosis [a:4, ROR (95% CI lower limit): 319.332 (93.475), PRR (X^2^): 319.294 (807.666), IC025: 5.807, EBGM (95% CI lower limit): 288.930]; Blood Ketone Body Present [a:22, ROR (95% CI lower limit): 315.408 (187.007), PRR (X^2^): 315.200 (4,405.402), IC025: 5.952, EBGM (95% CI lower limit): 2844.688]; Blood pH Abnormal [a:10, ROR (95% CI lower limit): 310.518 (143.327), PRR (X^2^): 310.425 (1,982.753), IC025: 5.893, EBGM (95% CI lower limit): 1038.315]; Balanitis Candida [a:13, ROR (95% CI lower limit): 302.782 (154.156), PRR (X^2^): 302.664 (2,535.362), IC025: 5.889, EBGM (95% CI lower limit): 1441.235] (Figure 4A). The corresponding System Organ Classes (SOC) were primarily focused on Infections and Infestations (24.30%), Investigations (19.63%), Reproductive System and Breast Disorders (14.49%), Renal and Urinary Disorders (7.01%), and Metabolism and Nutrition Disorders (6.54%) (Figure 4B).

### 3.5 Intersection analysis of SGLT-2 inhibitors

**Figure 5:**
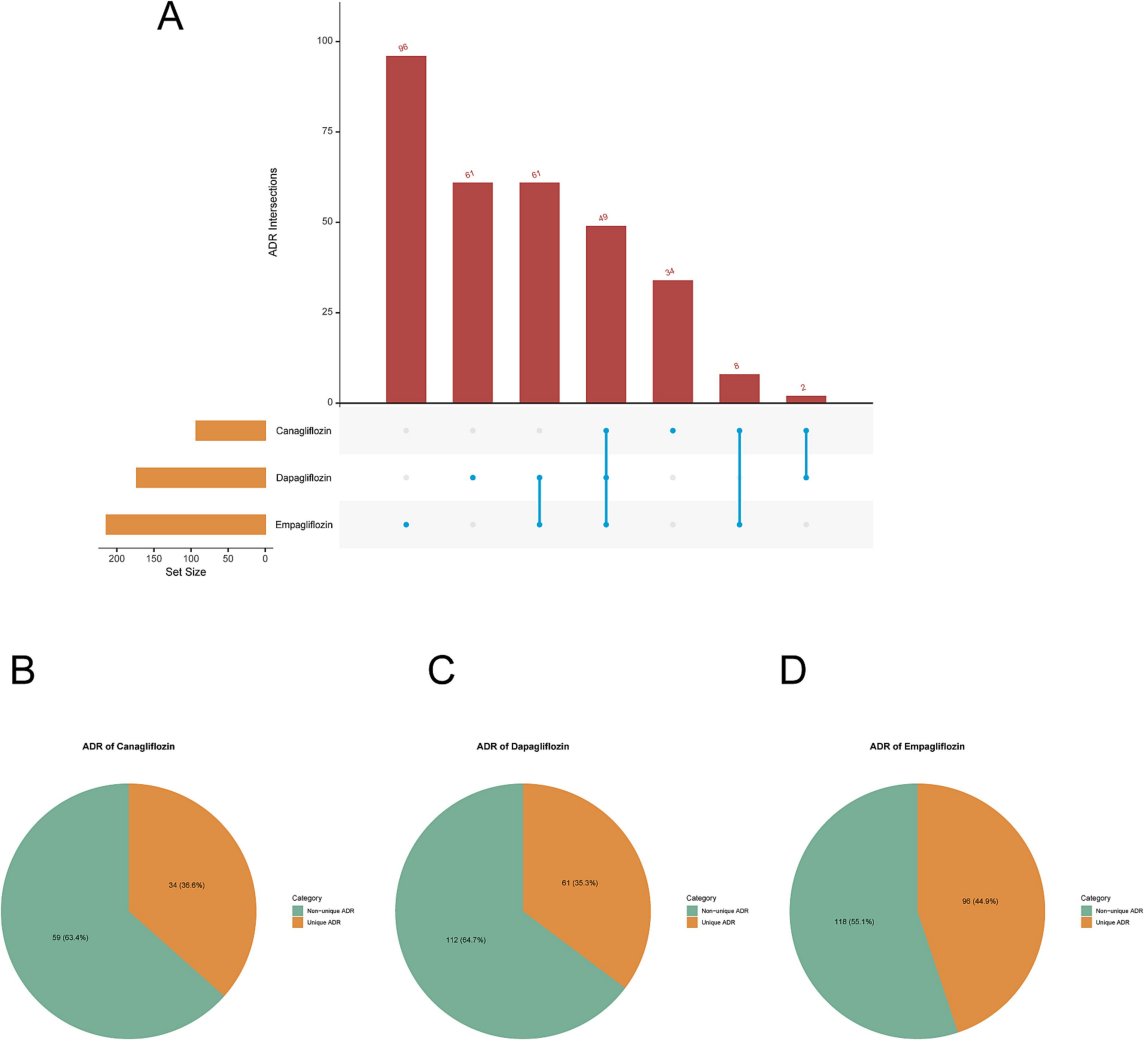
Intersection analysis. **(A)** UpSet plot visualization of positive ADRs intersection analysis among canagliflozin, dapagliflozin, and empagliflozin. **(B)** Canagliflozin positive unique and non-unique ADRs pie chart. **(C)** Dapagliflozin positive unique and non-unique ADRs pie chart. **(D)** Empagliflozin positive unique and non-unique ADRs pie chart.

Following intersection analysis among the adverse drug reactions (ADRs) of Canagliflozin, Dapagliflozin, and Empagliflozin subgroups, we identified a total of 49 common ADRs across the three SGLT-2 inhibitors (Figure 5A), including at the Preferred Term (PT) level: Abnormal Loss Of Weight, Amputation, Anion Gap Increased, Balanoposthitis, Blood Bicarbonate Decreased, Blood Ph Decreased, Candida Sepsis, Carbon Dioxide Decreased, Creatinine Renal Clearance Increased, Diabetic Gangrene, Dry Gangrene, Emphysematous Cystitis, Emphysematous Pyelonephritis, Escherichia Sepsis, Extremity Necrosis, Foot Amputation, Gangrene, Genital Burning Sensation, Genitourinary Tract Infection, Glomerular Filtration Rate Increased, Haematocrit Increased, Haemoglobin Increased, Heat Illness, Ketonuria, Ketosis, Lactic Acidosis, Leg Amputation, Necrotising Fasciitis, Oedema Genital, Pancreatitis Necrotising, Penile Pain, Penile Swelling, Penis Disorder, Phimosis, Polydipsia, Renal Abscess, Renal Pain, Systemic Candida, Testicular Pain, Thirst, Urinary Tract Infection Bacterial, Urinary Tract Infection Fungal, Urine Albumin/Creatinine Ratio Increased, Urine Ketone Body, Urine Ketone Body Present, Urine Odour Abnormal, Urine Output Increased, Urosepsis, Vulval Abscess. Among the 173 ADRs of Dapagliflozin, 61 (35.3%) were unique ADRs, while 112 (64.7%) were non-unique ADRs (Figure 5B). Empagliflozin had a high number of unique ADRs with 96 (44.9%) (Figure 5C), while Canagliflozin had a lower number of unique ADRs with only 34 (36.6%) (Figure 5D). It can be observed that the proportion of unique ADRs among the three is relatively close.

### 3.6 Multivariable logistic regression

**Figure 6:**
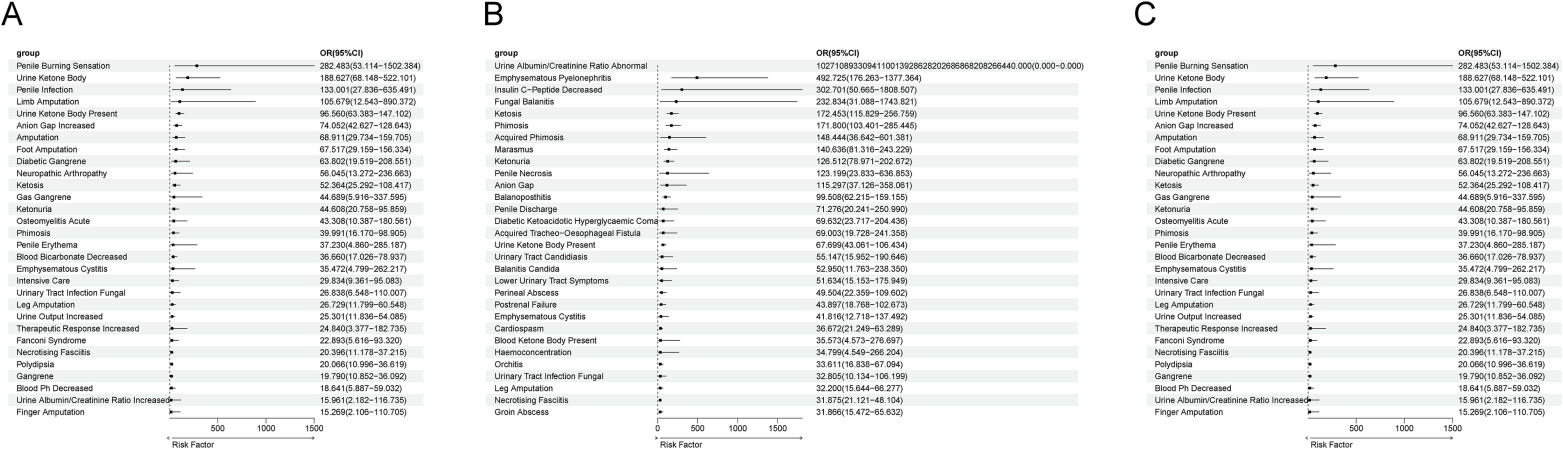
Multivariate logistic regression analysis of confounding factors including age, gender, and body weight. **(A)** Multivariate Logistic Regression Results for ADR of Empagliflozin. **(B)** Multivariate Logistic Regression Results for ADR of Dapagliflozin. **(C)** Multivariate Logistic Regression Results for ADR of Canagliflozin.

The results indicate that all drugs have a potential to promote ADRs. Among the 214 ADRs of Empagliflozin, multivariable logistic regression showed that Empagliflozin played an independent risk factor role in 161 ADRs (OR > 1 and *p* < 0.05), while in the remaining ADRs, the risk posed by Empagliflozin might be influenced by factors such as age and gender (OR > 1 and *p* > 0.05) (Figure 6A). Among the 173 ADRs of Dapagliflozin, Dapagliflozin played an independent risk factor role in 113 ADRs (OR > 1 and *p* < 0.05), while in the remaining ADRs, the risk posed by Dapagliflozin might be influenced by factors such as age and gender (OR > 1 and *p* > 0.05) (Figure 6B). Among the 93 ADRs of Canagliflozin, Canagliflozin played an independent risk factor role in 57 ADRs (OR > 1 and *p* < 0.05), while in the remaining ADRs, the risk posed by Canagliflozin might be influenced by factors such as age and gender (OR > 1 and *p* > 0.05) (Figure 6C).

### 3.7 Identification of positive ADRs for specific SGLT-2 inhibitors compared to other SGLT-2 inhibitors

The results indicate that among the shared ADRs for empagliflozin and dapagliflozin, 61 ADRs were not detected for canagliflozin. These include Acquired Phimosis, Anal Abscess, Anion Gap, Arterial Stenosis, Bacteriuria, Balanitis Candida, Bile Duct Cancer, Bladder Neoplasm, Blood Bicarbonate Abnormal, Increased Blood Creatinine, Blood Ketone Body Present, Breath Odor, Positive Urine Culture, Dermo-Hypodermitis, Diabetic Coma, Diabetic Ketoacidotic Hyperglycemic Coma, Abnormal Ejection Fraction, Fasciitis, Genital Abscess, Genital Erythema, Female Genital Infection, Male Genital Infection, Genital Pain, Genital Tract Inflammation, Groin Abscess, Groin Infection, Hydronephrosis, Hyperosmolar State, Decreased Insulin C-Peptide, Intertrigo, Leukocyturia, Increased N-Terminal Prohormone Brain Natriuretic Peptide, Necrotizing Soft Tissue Infection, Orchitis, Paraphimosis, Penile Discharge, Perineal Abscess, Perineal Cellulitis, Perirectal Abscess, Physical Deconditioning, Postrenal Failure, Prerenal Failure, Prostatic Abscess, Pyelitis, Pyuria, Respiratory Alkalosis, Scrotal Abscess, Scrotal Infection, Scrotal Swelling, Skin Candida, Starvation, Testicular Abscess, Testicular Swelling, Thrombotic Cerebral Infarction, Ureterolithiasis, Urethritis, Urinary Tract Candidiasis, Vulvovaginal Burning Sensation, Vulvovaginal Pain, and Wound Necrosis.

Among the shared adverse drug reactions (ADRs) of canagliflozin and empagliflozin, eight were not detected with dapagliflozin. These include Increased Blood Lactic Acid, Presence of Blood in Urine, Finger Amputation, Limb Amputation, Metabolic Surgery, plenel erythema, plenel infection, and Sudden Hearing Loss.

Additionally, within the shared ADRs of canagliflozin and dapagliflozin, two ADRs were not identified with empagliflozin: Intensive Care and Abnormal Skin Odor.

## 4 Discussion

SGLT-2 inhibitors are a new class of medications for treating type 2 diabetes that have gained widespread use in patients with a history of cardiovascular disease or kidney issues. However, their side effects, including acute kidney injury, increased risk of urinary and reproductive infections, diabetic ketoacidosis, fractures, and amputations, have become increasingly prominent. These ADRs are noted in FDA labeling and have garnered significant attention from clinicians. Nonetheless, overlooked potential ADRs continue to undermine the confidence of both healthcare providers and patients.

This study is the first to comprehensively explore the similarities and differences in ADRs among SGLT2 inhibitors based on FAERS data. An interesting observation was made: after reaching a peak in ADR reports in 2015, the number for canagliflozin quickly declined. The decline can be attributed to warnings issued by the FDA between 2015 and 2016 regarding the increased risk of fractures and lower limb amputations associated with canagliflozin (with incidence rates of 4.0 and 2.6%, respectively). The European product overview also mentioned the fracture risk. These concerns stem from the two-year CANVAS study, which found that canagliflozin treatment increased bone turnover rates and decreased total hip bone density compared to the control group (17). As the use of canagliflozin became more restricted, the use of empagliflozin and dapagliflozin increased.

This highlights that identifying drug ADRs can aid in adjusting treatment regimens to ensure safe medication use.

Moreover, the number of ADRs associated with empagliflozin and dapagliflozin has increased yearly. This comprehensive study identified 93 ADRs for canagliflozin, primarily related to infections and infections, investigations, reproductive system and breast disorders, surgical and medical procedures, and metabolism and nutrition disorders. We also found 173 ADRs associated with dapagliflozin, mainly in Infections and Infestations, Investigations, Reproductive System and Breast Disorders, Renal and Urinary Disorders, and Nervous System Disorders. In contrast, empagliflozin showed an even higher number of ADRs, totaling 214, significantly exceeding that of canagliflozin. These ADRs mainly concentrate on infections and infections, investigations, reproductive system and breast disorders, renal and urinary disorders, metabolism, and nutrition disorders. Interestingly, the common ADRs among the three SGLT-2 inhibitors were primarily focused on infections and infections, investigations, and reproductive system and breast disorders, as confirmed by FDA labeling. Notable examples include limb necrosis, gangrene, ketoacidosis, urinary tract bacterial infections, and urinary tract fungal infections.

In a study by Varshney et al., the risk of urinary and genital infections was compared between SGLT2 inhibitors (SGLT2i) and GLP-1 receptor agonists in elderly patients (65 and older) with type 2 diabetes. The results indicated that SGLT2i use was not associated with an increased risk of composite urinary and genital infections compared to other second-line glucose-lowering agents (18). Similarly, a large multicenter real-world study involving type 2 diabetes patients in Canada and the UK found that SGLT2i treatment did not increase the risk of urosepsis compared to DPP-4 inhibitors (19). However, these conclusions have faced scrutiny; Klaorat Prasongdee’s meta-analysis revealed that users of dapagliflozin (OR = 0.92; 95% CI, 0.51 to 1.67; *p* = 0.82; *I*^2^ = 0%) or canagliflozin (OR = 0.32; 95% CI, 0.03 to 3.63; *p* = 0.27; *I*^2^ = 17%) had a risk of urosepsis (20). Furthermore, cases of acute pyelonephritis following the initiation of dapagliflozin in individuals with bladder outlet obstruction raised concerns, and these adverse drug reactions (ADRs) were confirmed in this study.

Subsequently, we identified differential adverse drug reactions (ADRs) among SGLT2 inhibitors. Data indicate that type 2 diabetes mellitus (T2DM) may be associated with an increased risk of bladder cancer, with a meta-analysis showing a 35% increased risk in diabetic patients compared to healthy individuals (21, 22). Compared to non-diabetic patients, those with T2DM have a higher likelihood of developing bladder cancer [OR = 3.6, 95%CI:1.1–11.2]. However, this risk is commonly attributed to the disease itself. It wasn’t until insulin was found to be associated with an increased risk of breast cancer that scholars began to focus on the potential bladder cancer risk of SGLT2 inhibitors. Nevertheless, there has been clinical disagreement regarding the occurrence of bladder cancer with SGLT2 inhibitors. In a pooled study of dapagliflozin, 9 cases of bladder cancer were reported in 5,478 dapagliflozin-treated patients, compared to 1 case in 3,156 controls (23). Previously, Huilin Tang and colleagues, through a meta-analysis of several randomized controlled trials, suggested that SGLT2 inhibitors, especially empagliflozin, might increase the risk of bladder cancer (24), while Agata Ptaszynska and others (25) hold a different view. Currently, the hypothesis supporting the former conclusion posits that SGLT2 inhibitors might promote the growth of malignant cells through persistent glycosuria or chronically irritate the bladder epithelium through recurrent urinary tract infections, thereby increasing the risk of bladder cancer (26). In this study, we first confirmed that SGLT2 inhibitors indeed carry a risk of bladder cancer, and even bile duct cancer, with this risk being primarily concentrated in dapagliflozin and empagliflozin. Therefore, for high-risk cancer patients, selective medication should be considered, such as using lower doses or switching to other hypoglycemic drugs, and turning to canagliflozin. At the same time, it is necessary to strengthen regular monitoring and follow-up, including urinalysis and imaging examinations such as ultrasound, CT, to identify early symptoms for timely adjustments.

We also found ADRs not mentioned in the instructions, among which thrombotic cerebral infarction deserves close attention. Although SGLT2 inhibitors have been proven to have cardiovascular protective effects, and the incidence of major adverse cardiovascular events, cardiovascular mortality, heart failure, and all-cause mortality observed under SGLT2i treatment have all been reduced, non-fatal strokes unexpectedly increased by 30% (27). However, other evidence suggests that SGLT2is have no significant impact on ischemic events caused by atherosclerotic coronary artery disease in patients with type 2 diabetes (28). There is controversy over whether SGLT2is can cause these adverse events, and there is no evidence from large pharmacoepidemiologic studies or clinical trials. However, what we do know is that the osmotic diuretic effect of SGLT2 inhibitors may lead to hypovolemia, especially when used in combination with diuretics, and even dehydration due to excessive diuresis (29), which could lead to thrombotic events such as cerebral infarction. In addition, an increase in low-density lipoprotein (LDL) by SGLT2 inhibitors is also a possible explanation, but this increase has not shown significant differences between drug types (30), and in this study, the risk of thrombotic cerebral infarction is mainly concentrated in dapagliflozin and empagliflozin. Therefore, for patients with a history of thrombosis, it is recommended to start with a lower dose of these two drugs to assess tolerance and effectiveness. After starting treatment, regularly monitor the patient’s blood volume status, observe for signs of dehydration or hypovolemia, and encourage patients to drink an appropriate amount of water regularly. At the same time, monitor the levels of low-density lipoprotein (LDL) in the blood to assess the impact of SGLT2 inhibitors on lipid metabolism. In addition, cerebral infarction is also a rare and severe complication of DKA (31).

Additionally, we identified some controversial ADRs. Chen et al. (32) explored the correlation between SGLT2 inhibitors and cellulitis based on Mendelian randomization and found no causal relationship. However, this study was conducted without considering the population with diabetes mellitus (DM), who are known to be inherently at a higher risk of ulcers and infections. Moreover, SGLT2 inhibitors carry a risk of peripheral artery disease, thus the risk of cellulitis in the DM population with SGLT2 inhibitors cannot be ruled out. Furthermore, the study did not target specific SGLT2 inhibitor medications. In this study, aside from canagliflozin, empagliflozin and dapagliflozin were both associated with skin and subcutaneous tissue inflammation as well as perianal cellulitis, while no increased risk of perianal cellulitis was observed.

Although this study has integrated reliable information, such as only selecting reports submitted by healthcare professionals and limiting drugs labeled with “PS,” and conducted four signal detection methods, it is difficult to avoid limitations such as voluntary reporting and unclear causality. Further exploration of the mechanisms will help to improve the evidence chain.

## 5 Conclusion

In summary, adverse drug reactions (ADRs) induced by SGLT2 inhibitors are mainly concentrated in infections and disturbances, laboratory tests, and reproductive and breast diseases, specifically manifesting as urinary and reproductive system inflammation-related conditions, such as orchitis and testicular abscess, which is consistent with the information in the FDA label. However, this study also identified some ADRs that have not received widespread attention, including bladder cancer, bile duct cancer, and thrombotic cerebral infarction, which have not been detected in the use of canagliflozin. Therefore, before starting treatment, prescribers should be aware of whether patients have comorbidities in the above systems and monitor them during the treatment process to prevent disease exacerbation.

Furthermore, subsequent studies should focus on exploring the causal relationship between SGLT2 inhibitors and the induction of bladder cancer and thrombotic cerebral infarction. This will help develop drugs with fewer ADRs while ensuring efficacy. In addition, further mechanistic studies and large-scale clinical trials will help clarify the biological basis of these risks, providing safer guidance for clinical practice.

## Data Availability

All data generated or analysed during this study are included in this published article. Publicly available datasets were analyzed in this study. These data can be found at: https://www.fda.gov/.
